# Optimising corticosteroid injection for lateral epicondylalgia with the addition of physiotherapy: A protocol for a randomised control trial with placebo comparison

**DOI:** 10.1186/1471-2474-10-76

**Published:** 2009-06-24

**Authors:** Brooke K Coombes, Leanne Bisset, Luke B Connelly, Peter Brooks, Bill Vicenzino

**Affiliations:** 1 The University of Queensland, Division of Physiotherapy, School of Health and Rehabilitation Sciences, St Lucia, QLD, 4072, Australia; 2 Griffith University, School of Physiotherapy and Exercise Science, Gold Coast Campus, QLD, 4222, Australia; 3 Royal Brisbane & Women's Hospital, Physiotherapy Department, Herston, QLD, 4006, Australia; 4 The University of Queensland, Australian Centre for Economic Research on Health (ACERH), Centre of National Research on Disability and Rehabilitation Medicine (CONROD) and School of Economics, Level 3 Mayne Medical School, Herston Rd, Herston, QLD, 4006, Australia; 5 The University of Queensland, Faculty of Health Sciences, Edith Cavell Building, Royal Brisbane and Women's Hospital, Herston, QLD, 4006, Australia

## Abstract

**Background:**

Corticosteroid injection and physiotherapy are two commonly prescribed interventions for management of lateral epicondylalgia. Corticosteroid injections are the most clinically efficacious in the short term but are associated with high recurrence rates and delayed recovery, while physiotherapy is similar to injections at 6 weeks but with significantly lower recurrence rates. Whilst practitioners frequently recommend combining physiotherapy and injection to overcome harmful effects and improve outcomes, study of the benefits of this combination of treatments is lacking. Clinicians are also faced with the paradox that the powerful anti-inflammatory corticosteroid injections work well, albeit in the short term, for a non-inflammatory condition like lateral epicondylalgia. Surprisingly, these injections have not been rigorously tested against placebo injections. This study primarily addresses both of these issues.

**Methods:**

A randomised placebo-controlled clinical trial with a 2 × 2 factorial design will evaluate the clinical efficacy, cost-effectiveness and recurrence rates of adding physiotherapy to an injection. In addition, the clinical efficacy and adverse effects of corticosteroid injection beyond that of a placebo saline injection will be studied. 132 participants with a diagnosis of lateral epicondylalgia will be randomly assigned by concealed allocation to one of four treatment groups – corticosteroid injection, saline injection, corticosteroid injection with physiotherapy or saline injection with physiotherapy. Physiotherapy will comprise 8 sessions of elbow manipulation and exercise over an 8 week period. Blinded follow-up assessments will be conducted at baseline, 4, 8, 12, 26 and 52 weeks after randomisation. The primary outcome will be a participant rating of global improvement, from which measures of success and recurrence will be derived. Analyses will be conducted on an intention-to-treat basis using linear mixed and logistic regression models. Healthcare costs will be collected from a societal perspective, and along with willingness-to-pay and quality of life data will facilitate cost-effectiveness and cost-benefit analyses.

**Conclusion:**

This trial will utilise high quality trial methodologies in accordance with CONSORT guidelines. Findings from this study will assist in the development of evidence based practice recommendations and potentially the optimisation of resource allocation for rehabilitating lateral epicondylalgia.

**Trial registration:**

Australian New Zealand Clinical Trials Register ACTRN12609000051246

## Background

Lateral epicondylalgia (LE), also known as 'tennis elbow', is a musculoskeletal disorder characterised by pain over the lateral humeral epicondyle associated with gripping or manual tasks that require manipulation of the hand. With an annual incidence of 1–3% within the general population [1-5], LE is a common condition that significantly impacts on the individual and society. Corticosteroid injection and physiotherapy are commonly prescribed conservative treatments for this condition. Systematic review evidence has found corticosteroid injection provides superior short term benefits [6-9], but recent studies suggest poorer clinical outcomes in the longer term [10,11]. One aspect of this poorer outcome is the significantly larger recurrence rates that have been reported following corticosteroid injection (72%) as compared to physiotherapy (8%) and adoption of a wait and see policy (10%) [10]. These late adverse outcomes are of concern to both patients and their doctors.

Despite their regular prescription, there is a critical need to evaluate the therapeutic and adverse effects of the corticosteroid injection compared to placebo. The anti-inflammatory mode of action of corticosteroid medication has been questioned by evidence of an absence of classic inflammatory mediators in this condition [12,13]. Local injection of corticosteroid may mediate its effect through alterations in the release of noxious chemicals or inhibition of collagen, extracellular matrix proteins and granulation tissue [12,14]. However, whether these effects are ultimately clinically beneficial or harmful in the long term is not known.

From a clinical perspective, practitioners frequently emphasise the importance of an active rehabilitation program, either in isolation or in combination with injection. In a recent study, [10] physiotherapy comprising specific elbow manipulation combined with a progressive exercise program was found to be as effective as the corticosteroid injection at 6 weeks, while superior to injection at 12 weeks. When considering the overall effect over the entire 12-month follow-up period, calculated by area under the curve, physiotherapy was superior to both corticosteroid injection and a wait and see policy [10]. Combination of injection with physiotherapy modalities has only been evaluated in two small studies [15,16]. One reported that corticosteroid injection did not provide significant improvement in outcome when added to a program of ice massage and physiotherapy prescribed exercises (monitored at 3–4 weekly physiotherapy sessions) for patients with LE of less than four weeks duration [16]. The authors recommended that rehabilitation be the first line of treatment. The other study found no significant effect of a progressive graduated exercise program (number of physiotherapy sessions not specified) when added to injection, however this study was underpowered (small sample size), reported a high drop-out rate and did not assess long term outcomes (only reported a 7 week follow up) [15]. Hence, there is a high likelihood that there was an unacceptable Type II error rate with this study.

Increasingly, the discovery of efficacious treatments needs to be considered in the context of their costs. Research into the cost effectiveness of corticosteroid injections, physiotherapy or just adopting a wait and see policy for LE demonstrated no significant cost-effectiveness differential [17]. This project will provide economic evaluation of the costs to relative benefits of the addition of physiotherapy to injection. Computation of cost-effectiveness ratios will enable a comparison of these interventions with the alternatives, and to compare results with others studies that have been subjected to economic analysis. In addition, the collection of willingness-to-pay data via a contingent valuation study, will enable the computation of net present value differences between the benefits and costs of the intervention. The latter approach will determine whether or not, in an absolute sense, the intervention is worthwhile, on economic grounds.

In summary, the following hypotheses will be evaluated in order to address the aims of the study: (1) Addition of physiotherapy to an injection when compared to injection alone will improve the long term efficacy and reduce the recurrence rates; (2) The combined corticosteroid and local anaesthetic injection will be superior to that of saline injection in the short term but not long term; (3a) The benefits gained by adding physiotherapy to injection outweigh the costs associated with the injection alone; and (3b) the cost-effectiveness of the combined therapy is superior to the cost-effectiveness of injection alone.

## Methods/design

**Figure 1:**
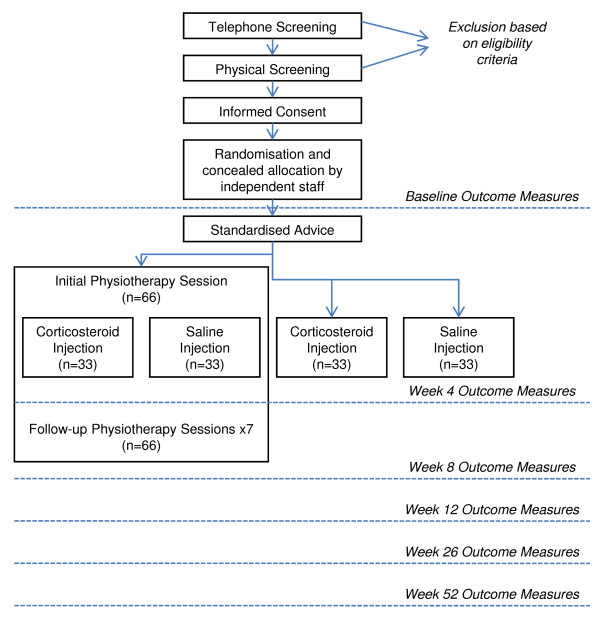
**Process of recruitment, randomization to treatment, treatment provision, and outcomes assessment**.

A clinical trial with a 2 × 2 factorial design will be used to study the above aims in a similar manner to a previous study of shoulder conditions [18]. The two levels of the two main effects of injection and physiotherapy will be combined to constitute four treatment groups (1) corticosteroid injection alone, (2) saline injection alone, (3) corticosteroid injection and physiotherapy; (4) saline injection and physiotherapy. An overview of the study protocol is provided in Figure 1. Ethical approval has been obtained from the Medical Research Ethics Committee at the University of Queensland.

### Eligibility Criteria

Volunteers will be self-selected from the Brisbane and surrounding regions using a comprehensive public notification strategy (e.g., advertising and media releases). A two stage screening process will be used, comprising a telephone interview followed by a clinical examination, to determine eligibility for the study (Table 1) and familiarise the participant with testing procedures. Following a detailed explanation of the study protocol, eligible participants will be invited to return on a separate occasion to complete consent documentation and baseline outcome measurements.

**Table 1:** Eligibility criteria

**Inclusion criteria**
Unilateral elbow pain for longer than six weeks
Pain severity equal or greater than 30 mm on a 100 mm visual analogue scale
Pain over the lateral humeral epicondyle provoked by at least two of: gripping, palpation, stretching of forearm extensor muscles and resisted wrist or middle finger extension
Reduced pain-free grip force
Age between 18–70 years
An acceptable understanding of written and spoken English
Willingness to comply with treatment and follow-up assessments


**Exclusion criteria**

Injection within the preceding 6 months
Course of exercise based physiotherapy program within the preceding 3 months
Concomitant neck or other arm pain that has prevented participation in usual work or recreational activities or necessitated treatment within the last 6 months
Evidence of other primary sources of lateral elbow pain including: exacerbation of elbow pain with neck movements or manual examination; pain localised over the radiohumeral joint, sensory disturbance in the affected hand
History of fractures within the preceding 10 years, elbow surgery, malignancy, inflammatory or arthritic disorder
Any medical condition which may contraindicate injection or exercise prescription
Pregnant or breastfeeding

### Interventions

Interventions will be administered in a primary care setting by one medical practitioner and one physiotherapist (if allocated) in a manner that is considered best practice. All practitioners will receive training to ensure treatments are provided in accordance with the following study protocol.

### Injection

A routine clinical examination will be conducted by the treating medical practitioner prior to injection of either (1) 1 ml Triamcinolone Acetonide (10 mg/ml) (Kenacort-A 10) with 1 ml Lignocaine (1%) [Corticosteroid injection] or (2) 0.5 ml Isotonic Saline (0.9%) [Saline injection]. Injection will be administered into the most palpably tender point(s) in the region of the lateral epicondyle, consistent with previous studies [10,16,19,20]. The syringe will be drawn up and administered with the participant unable to view its contents to facilitate participant blinding. In addition, standardised post-injection information will be provided verbally and in the form of a printed pamphlet based on published recommendations [10,16,21]. Rest from all strenuous activity for 1–2 weeks following injection will be strongly recommended, followed by gradual return to normal activities. Participants will be instructed to avoid aggressive return to activities even if substantial relief is obtained, to minimise potential recurrence of their symptoms. All participants will be warned of normal post-injection responses and to inform their doctor if there is any suggestion of infection or other adverse events. All adverse reactions will be managed by a committee chaired by the chief investigator (BV).

### Physiotherapy

A standardised physiotherapy treatment protocol has been devised based on a recently evaluated program [10]. Eight, 30 minute physiotherapy sessions will be provided by a post-graduate qualified physiotherapist over an 8 week period. The initial physiotherapy consultation will be scheduled prior to injection to allow baseline measurement and familiarisation with the program. The primary rationale for physiotherapy rehabilitation will be relief of pain, restoration of motor function and facilitation of tendon healing [22,23]. A pragmatic multimodal program comprising education, manipulation and therapeutic exercise will be used in conjunction with a home exercise program to address these aims.

#### Manual Therapy

**Figure 2:**
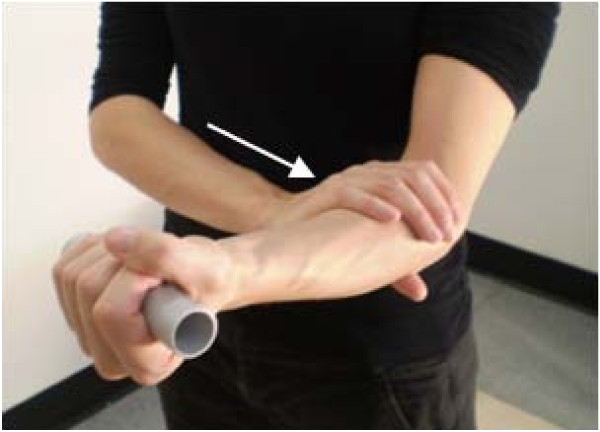
**Retraining of gripping using self-applied lateral (Mobilisation with Movement) glide**.

Specific elbow manipulation techniques known as Mobilisation with Movement (MWM) will be applied, as described by Vicenzino (2003) [23], based on evidence of their immediate effects on pain and improved pain-free grip force; [24,25]. A hydraulic grip dynamometer will be used to measure baseline pain-free grip force and the effect of the following glides – lateral elbow glide, postero-anterior radioulnar glide and de-loading of the common extensor origin [23]. With the participant's arm supported in elbow extension and pronation, the therapist will sustain a glide while the participant slowly performs a pain-free grip over approximately 6 seconds. If substantial improvement in pain-free grip force is noted with the glide application, the technique may be repeated 6–10 times during a single treatment session. If successful, self-treatment techniques (Figure 2) will be taught to the participant using dynamometer feedback in the clinic.

#### Therapeutic Exercise

**Figure 3:**
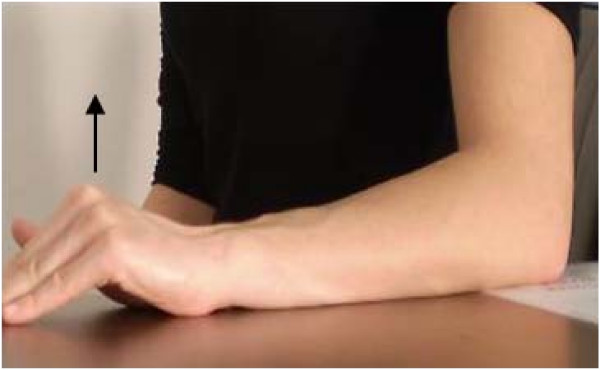
**Sensorimotor exercise for retraining of isolated wrist extension, with emphasis on avoiding metacarpophalangeal extension**.

**Figure 4:**
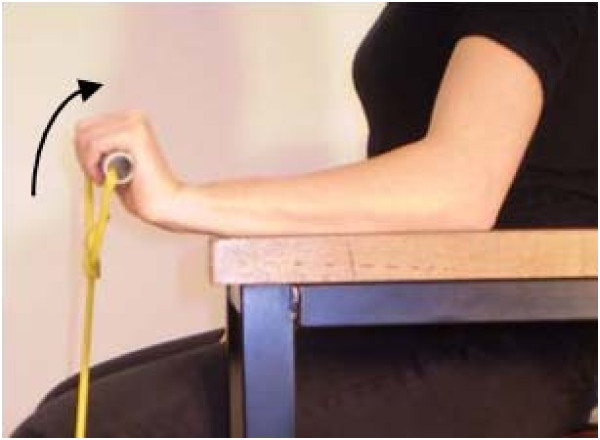
**Progressive resistance exercise for wrist extensors using Theraband™**.

A comprehensive exercise program will be used to primarily address motor impairments, but also to address pain and stimulate tendon remodelling [22]. Three main groups of exercises will be pragmatically prescribed: (1) Sensorimotor retraining of gripping and forearm movements (Figure 3) and posture correction will be commenced early in the physiotherapy intervention. These exercises are based on identified motor control deficits [26-28] and previous 'Occupational training' exercises for LE [29]. (2) Progressive resistance exercise for the wrist extensors will be prescribed based on identified strength deficits in LE [30,31] and proposed effects of exercise on tendon remodelling [32,33]. Combined concentric and eccentric exercise will be performed, given insufficient evidence for use of eccentric over concentric modes in LE [34,35]. Thera-Band™ tubing of varied resistance levels with an attached handle will be used to provide progressive resistance (Figure 4). Restriction of painful segments of range will be recommended in early rehabilitation, with progression to maximal positions of loading involving elbow extension and forearm pronation as tolerated [36]. (3) Exercises geared towards general arm strengthening will be performed with free weights or weight bearing anti-gravity exercise to address known proximal and bilateral strength deficits found in LE sufferers [31]. Progression of these exercises to include work or sport-specific exercises may occur later in rehabilitation.

Physiotherapists will prescribe exercises based on the participant's capabilities at any given session to allow for optimal exercise volume and load setting without exacerbating pain [23]. The overriding rule for all exercise is that pain should not be provoked during or after exercise, including avoidance of delayed onset muscle soreness. Exercises will be performed in a slow manner with sufficient rest between sets to allow recovery, and correct form and posture emphasised throughout. Supervision of the home program at the commencement of every session and monitoring of exercise diaries by the treating practitioners will be used to facilitate program adherence and emphasise the importance of the exercise and self-treatment program.

### General information for all participants

All participants will receive standardised advice regarding activity modification and pain management at the commencement of the study, in the form of a printed pamphlet and verbal assessment of their understanding of its contents. They will be advised that complete rest is detrimental to chronic musculoskeletal disorders and activity that does not cause elbow pain should be encouraged. Participants will be requested to refrain from seeking not-per-protocol treatments, however analgesic medications and elbow braces will be permitted throughout the study if the participant feels they are needed. Participants will be questioned at each measurement session regarding usage of not-per-protocol treatments, including brace use. On completion of the trial, participants will complete an exit questionnaire and rate their satisfaction with treatment(s).

### Outcome assessment

Participants will be evaluated by a blinded assessor at baseline, 4, 8, 12, 26 and 52 weeks following randomisation. The 4 and 8 week time points will be used primarily to investigate the short term effects of corticosteroid injection compared to saline injection and of addition of physiotherapy to injection respectively. The 52 week time point will provide a primary end point for study of all long term effects. The data from the other measurement points (12–52 weeks) will be used to derive indices for recurrence rates. These time points have been chosen on the basis of a previous study which noted deterioration in the corticosteroid injected group during this time period [10].

Socio-demographic information will be collected at baseline including age, gender, employment nature, smoking status, body mass index and physical activity status. Information will be sought regarding the nature, duration and onset of LE symptoms, previous treatment and presence of concomitant neck or arm pain. The following outcome measures will be collected at baseline assessment and at follow-up evaluations as outlined in Table 2.

**Table 2:** Outcome measures used at baseline and follow-up interviews

**Outcome Measures**	**Time Point (weeks)**	
	
	**0**	**4, 8, 12, 26 & 52**
Global Perceived		x
Improvement		
Resting/Worst Pain (VAS)	x	x
Pain/Disability (PRTEE)	x	x
Quality of Life (EuroQol)	x	x
Anxiety/Depression (HADS)	x	
Kinesiophobia (Tampa)	x	
Pain-free Grip Force	x	x
Pressure Pain Threshold	x	x
Adverse events		x
Willingness-to-pay	x	
Costs		4 randomly allocated time points

### Primary Outcomes

#### Global perceived improvement

The participant's self-perceived level of improvement will be measured with a 6-point Likert scale with categories: completely recovered, much improved, improved, no change, worse and much worse [10,20]. The participant will be asked at each follow-up outcome assessment to rate the change in their elbow condition since their commencement in the study. A similar retrospective assessment scale has been shown to be more sensitive to change than serial visual analogue scale measures and more correlated with patient's satisfaction with change [37].

#### Success

Success will be derived from the global perceived improvement scale as per previous work [10,20]. The categories 'completely recovered' and 'much improved' will be dichotomised as 'success', while the other categories ('much worse' to 'improved') will be collapsed to represent 'no-success'.

#### Recurrence

Recurrence will primarily be defined as occurring when a participant rates a success at 4 or 8 weeks and a no-success beyond 8 weeks on the global perceived improvement scale [10]. This definition of recurrence which evaluates the pattern of recurrent events over time has been used in previous studies of LE [10,20,38]. In addition, participants will be questioned using a semi-structured interview at 12, 26 and 52 weeks about their experience of symptom aggravation and possible reasons for their recurrence. This data will enable better understanding of recurrences and best practice measurement of recurrence rates in future analyses and trials.

### Secondary outcome measures

#### Pain severity

Two visual analogue scales (VAS) anchored by 'no pain' (0 mm) and 'worst imaginable pain' (100 mm) will be used measure the severity of participants' resting pain and worst pain experienced during the preceding week. The VAS is considered the most sensitive of all pain rating scales and has been specifically evaluated in the LE population with high test-retest reliability (r = .89) and moderate correlation with pain-free grip (r = .47) [39].

#### Patient-rated Tennis Elbow Evaluation

The Patient-rated Tennis Elbow Evaluation (PRTEE) questionnaire will provide a standardised quantitative assessment of pain and functional disability. It has been shown to have excellent test-retest reliability (r = .93) and good correlation with other functional scales including the Disability of Arm and Shoulder (DASH) questionnaire (r = .87) in the LE population [40]. Questions are scored on an 11-point Likert scale, with calculation of separate subscales for pain and function and a total score, ranging from 0 (no pain and no functional disability) to 100 (worst imaginable pain with a very significant functional disability) [40].

#### Pain-free grip force

PFG force is well established as a highly reliable (ICC >.97) and convenient clinical assessment tool, which correlates more strongly with disability and perceived improvement than maximal grip strength in LE populations [41-43]. PFG force will be measured using a digital grip dynamometer with variable handle position (MIE, Medical Research, UK). The participant will be positioned in supine with the tested elbow in relaxed extension and pronation [6]. The participant will be instructed to maximally squeeze the dynamometer on the unaffected side. On the affected side, the participant will be asked to grip the dynamometer at the same rate as the unaffected side but to stop the instant pain is experienced. The average of three repetitions with 20 second rest intervals will be used in further analyses.

#### Pressure pain threshold

Pressure pain threshold (PPT) will be used as quantitative measure of mechanical hyperalgesia over the lateral epicondyle as per previous studies [10,20]. Pressure will be applied using a digital algometer with a probe size of 1 cm^2 ^(Somedic AB, Farsta, Sweden) at a rate of 40 kPa/s until the first sensation of pain is recorded. Triplicate recordings will be taken at each follow-up assessment and the mean values used for analysis. Measurements will be performed by the same blinded assessor (BC) as reliability has been found to be reduced between observers (r = .72–.98) [41].

#### The Hospital Anxiety and Depression Scale

The Hospital Anxiety and Depression Scale (HADS) will be used to identify and quantify the two most common forms of psychological disturbances – anxiety and depression [44]. It has been demonstrated to be an appropriate measure in musculoskeletal pain [45], with evidence of elevated levels of both anxiety and depression in the LE population [46]. HADS consists of 14 items which are independent of somatic symptoms and divided into two subscales for anxiety and depression. Each item is rated on a four point scale, with scores of >11 on either subscale considered to be a significant (probable or definite) case of psychological morbidity, whereas scores of 8–10 represent borderline (possible or doubtful) case and 0–7 normal (non-case) [45].

#### Tampa Scale for Kinesiophobia

The Tampa Scale for Kinesiophobia (TSK) will be administered at baseline to assess the degree of kinesiophobia, also known as fear of movement or (re)injury [47]. While it has not been studied in the LE population, previous study of non-traumatic complaints of the neck and arm found positive associations between kinesophobia and disability and co-morbidity of musculoskeletal complaints [48]. The adjusted version of this scale (TSK-AV) will be used due to reported improved factor structure [48]. Each of the 13 items are scored on a 4-point Likert scale giving a total score ranging from 13 to 52, with higher scores indicating greater kinesiophobia.

#### Health-related quality of life

The EuroQol EQ-5D instrument will be used to measure health-related quality of life, expressed as utility values ranging from 0 to 1, where 1 represents perfect health[49]. The utility weights captured by these preferences will enable the derivation of the Quality Adjusted Life Years (QALY) for each intervention and will be used in cost-utility analyses. One advantage of using this instrument is that results of this study can be compared with those of a previous trial which compared corticosteroid injection, physiotherapy and a wait and see policy for LE [17].

#### Costs

Cost data will be collected from a societal perspective, meaning that an attempt to capture all of the costs, regardless of which party or parties incur them, will be made. It will include a) direct health care costs, such as visits to doctors, therapists, investigations and prescribed medication; b) direct non-health care costs, such as costs of over-the-counter medication, hours of paid and unpaid household help, transportation and other out-of-pocket expenses; c) indirect costs, such as absence from work, housekeeping and other daily activities [50]. Participants will be asked to recall their out-of-pocket expenses incurred over the preceding month during a structured telephone interview by a research assistant not involved in outcome measurement. The initial interview date will be randomised by a computer-generated number sequence to 4, 8 or 12 weeks following commencement of the study and then subsequent interviews performed at three monthly intervals. This method of sampling was chosen to provide sufficient information while minimising the time commitment required of participants [51]. In addition, participants will be asked to provide consent for their Medicare data on in-hospital, out-of-hospital and pharmaceutical services to be provided by Medicare Australia. These data will capture expenditures incurred under Australia's universal, compulsory health care financing scheme, Medicare. This includes all expenditures incurred for general practitioner and specialist services, as well as pharmaceuticals that are listed on the Pharmaceutical Benefits Scheme.

#### Willingness-to-pay

A contingent valuation study will be conducted to elicit an individual's willingness-to-pay (WTP) for relief of the symptoms of LE. The contingent valuation approach results in monetary valuation of the benefits produced by a health program or health state [52]. An advantage of this approach is it allows valuation of transitory health states, which may be important given previous trials suggest considerable temporal variance in the effectiveness of interventions for LE [10]. Participants will be questioned at baseline assessment regarding the greatest amount they are willing to pay, based on the following hypothetical scenario: "*Suppose that your current elbow pain will persist over the next 12 months if untreated. Imagine that there is a new treatment that is quick and non-invasive. If it works, it will provide an immediate and complete cure of your tennis elbow. However, this treatment only works on 1 in 2 people. The treatment is not covered by Medicare or private insurance*." Bias will be minimised by using both a bidding game approach and a binary response approach. The bidding game approach involves exposing respondents to repeated bids and identifying the point of indifference between those bids. In the binary response approach, the respondent is asked to respond to only one bid. In both approaches, four bids ($50, $650, $1250 and $1850 in Australian Dollars) will be randomly allocated to respondents. The randomisation of bids is a further measure to reduce the bias that may be generated by the choice of a starting bid [53]. Specifically, it is known that high (low) starting bids may lead to higher (lower) WTP valuations. The starting bids will be based on the mean ± 2 standard deviations derived from a similarly designed pilot study of WTP in LE participants.

#### Adverse events

All adverse events, defined as any negative or unwanted reactions to intervention, will be recorded. These will include pain (lasting longer than 7 days), skin pigmentation changes, subcutaneous atrophy or any other reported physical discomfort. As well as the adverse effects committee (chaired by BV) that will manage adverse reactions at the time of the adverse reaction, we will administer a questionnaire at 8 and 52 weeks to record all such patient identified events. This will capture additional ill effects not reported at the time of their occurrence. These are likely to be minor aggravations in symptoms. The questionnaire will use open-ended questions and will be placed in a sealed envelope to be opened by a research assistant not involved in outcome measurement. The intervention-specific incidence of each adverse event will be computed.

### Randomisation and allocation

Participants will be randomly allocated to groups by concealed allocation. The Queensland Clinical Trials Centre, an independent off-site body, will be responsible for generating the computerised randomisation schedule. An independent research officer will administer the schedule and perform all communication between participants and treating practitioners. Randomisation will be stratified according to baseline pain severity, as it has been found to be a strong prognostic indicator for LE [54]. The mean VAS pain score reported in a previous clinical trial (57.5 mm) [10] will be used to classify participants into high and low baseline pain.

### Sample size

The primary aim of this project is to detect a clinically important difference between injection alone and physiotherapy plus injection. It is estimated that 120 participants will be required to detect a 25% difference between physiotherapy and no physiotherapy (α = 0.05, β = 0.2), assuming a success rate of 27% in the least significant group. This difference is based on previous work and is clinically meaningful [20]. An extra 10% will be recruited to allow for conservative loss to follow-up, bringing the total participants to 132. The power calculation is based on previous findings that injection and physiotherapy are both effective interventions [10,20] and that the combination of the two will be additive, but not synergistic in effect.

### Planned Data analysis

#### Clinical Efficacy

All analyses will be conducted on an intention to treat basis by an investigator who is blind to group allocation. The outcomes measured at 4, 8, 12, 26 and 52 weeks will be used to generate efficacy and recurrence indices that will be analysed using linear mixed and logistic regression models [10]. Baseline scores for each dependent variable will be entered as a covariate, relevant participant characteristics entered as a random effect and treatment conditions and time as fixed factors. Variables such as age, gender, duration of condition, nature of employment will be included as covariates in the analysis, if found to significantly influence outcomes over time. Regression diagnostics will be used to check for normality of the measures and homogeneity of variance where appropriate. Alpha will be set at 0.01 to compensate for the possible increase in type I error rates that may result from multiple testing. The dichotomous measures of success and recurrence will be analysed using relative risk, and numbers needed to treat in order to provide a meaningful indicator of treatment efficacy to practitioners.

#### Health Economics

Participants' utility weights will be derived from EQ-5D responses. These QALY computations will be used along with cost data to enable cost-effectiveness analyses to be undertaken. As Australian weights for the EQ-5D are currently unavailable, we propose to use the available scoring algorithms for the UK and NZ to compute QALY estimates, with the variation between results forming the basis for a cost-per-QALY sensitivity analysis [49]. Capturing participants' WTP for treatment will provide an alternative measure of the marginal benefits associated with intervention and will be used in cost-benefit analyses. Care will be taken to avoid double counting the benefits estimated via the contingent valuation approach. The distribution of the WTP data will be analysed to examine not only the distribution of the responses, but also the effect of the starting bid and of respondent income on WTP. Modern statistical procedures, such as bootstrapping, will be used to assess the uncertainty surrounding costs and effects.

## Discussion

The basis for a combined approach of injection and physiotherapy is twofold. First there is the evidence of a rapid improvement (3 weeks) following corticosteroid injections [10,20] that is followed by higher recurrence rates and a relative delay in recuperation in the long term (> 6 weeks) [10,11]. Second, is the comparable effect of physiotherapy to corticosteroid injections at 6 weeks and the relative superiority of physiotherapy, in terms of lower recurrence rates, after that point. In addition, there is evidence of a beneficial effect of preventing chronicity following an eight week exercise program in chronic LE patients, the majority of whom had not responded to local corticosteroid injection [29,55]. Improved forearm strength has been reasoned to protect against chronicity and recurrence [29,56].

Use of a placebo intervention coupled with blinding is intended to prevent bias resulting from non-specific effects associated with those receiving the intervention (placebo effects) [57]. Local anaesthetic injection has been utilised as a placebo comparison [58,59], however this medication may be in part responsible for short term analgesic effects. Normal saline, in comparison, is considered to have no, or very little, therapeutic effect. In this study, a small volume (0.5 ml) of normal saline will be injected to minimise potential mechanical effects of a volume of fluid on local structures.

This study adheres to the CONSORT statement for randomised controlled trials [57,60]. Randomisation of participants by concealed allocation will be performed, as this feature is known to minimise bias [61]. Blinding strategies will be employed such that the participant and assessor are blind to the injection content (double blinding). The nature of the physiotherapy program means that blinding of patients and practitioners is not possible, however the assessor will be blinded to the allocation of physiotherapy (single blinding). Success in blinding of injection content will be evaluated at 8 and 52 weeks by both participant and assessor. This trial uses outcome measures that have established reliability and validity to enhance the quality of the outcomes and facilitate comparison with studies of LE and other musculoskeletal conditions. The statistical analysis will be conducted on an intention-to-treat basis. The influence of group allocation on the utilisation of not-per-protocol treatments will also be evaluated, in order to fully describe patient behaviours in a realistic context in which there are more than the study's interventions available. This study has been designed to optimise applicability to the clinical setting so that practitioners will be able to use the data in their day-to-day management of LE. For example, participants will be included on explicit clinical diagnostic criteria, interventions will be delivered in primary care settings and many of the outcome measures can be readily used in clinical practice.

It is becoming increasingly important to determine the costs relative to the benefits, measured either in monetary values or gains in health-related quality of life, of the combined injection and physiotherapy approach. The inclusion of an economic evaluation in this trial provides a basis for considering the economic arguments for investments in the interventions of interest here, and their comparison to other health sector interventions. The measurement of costs from a societal perspective encompasses those costs incurred by the patient and his/her family, private and public sector payments and productivity losses [53]. Evaluation of the value of improved health will be measured using physical measures of health-related quality of life (e.g., QALYs) and WTP measures. The range of measures that will be used in this study provides us with considerable flexibility to examine the economic impacts of the interventions.

## Conclusion

An effective treatment strategy that provides rapid alleviation of LE and that is maintained in the long term is needed. This project is designed to provide high quality evidence evaluating the ability of physiotherapy to augment corticosteroid injection in the treatment of LE. It will do this by studying the clinical notion that the combined approach is preferred to that of injection alone. Additionally, it will provide further insight into the therapeutic basis of corticosteroid injection by comparison to a placebo injection.

## Competing interests

The authors declare that they have no competing interests.

## Authors' contributions

BV, LB, PB, and LC participated in the conception and design of this trial and are the chief investigators on the NHMRC grant # 511238. BC, LB, LC and BV were responsible for writing this manuscript. All authors read and approved the final manuscript.

## Pre-publication history

The pre-publication history for this paper can be accessed here:


